
JOURNAL INFORMATION
==============================
NLM Title Abbreviation: Aging Cell
Journal ID: Aging Cell
NLM Journal ID: 101130839
Journal ID: ACEL

Aging Cell

ISSN: 1474-9718
EISSN: 1474-9726
Publisher: Wiley

ARTICLE INFORMATION
==============================
PMCID: PMC11474303
DOI: 10.1111/acel.13881
Article ID: ACEL13881
Article version: 1
Subjects: Issue Information, Issue Information

Issue Information

Electronic publication date: 2024 Jun 11
Publication date: 2024 Jun
Volume: 23
Issue: 6
Electronic Location ID: e13881
Copyright: Copyright © 2024 The Anatomical Society and John Wiley & Sons Ltd
License: This is an open access article under the terms of the http://creativecommons.org/licenses/by/4.0/ License, which permits use, distribution and reproduction in any medium, provided the original work is properly cited.
License URL: https://creativecommons.org/licenses/by/4.0/

Figure count: 0
Table count: 0
Page count: 3

source-schema-version-number: 2.0
cover-date: June 2024
details-of-publishers-convertor: Converter:WILEY_ML3GV2_TO_JATSPMC version:6.4.9 mode:remove_FC converted:15.10.2024

==============================

### Aging Cell

ISSN 1474-9718

**Volume 23**

**Issue 6**

**June 2024**

Open Access

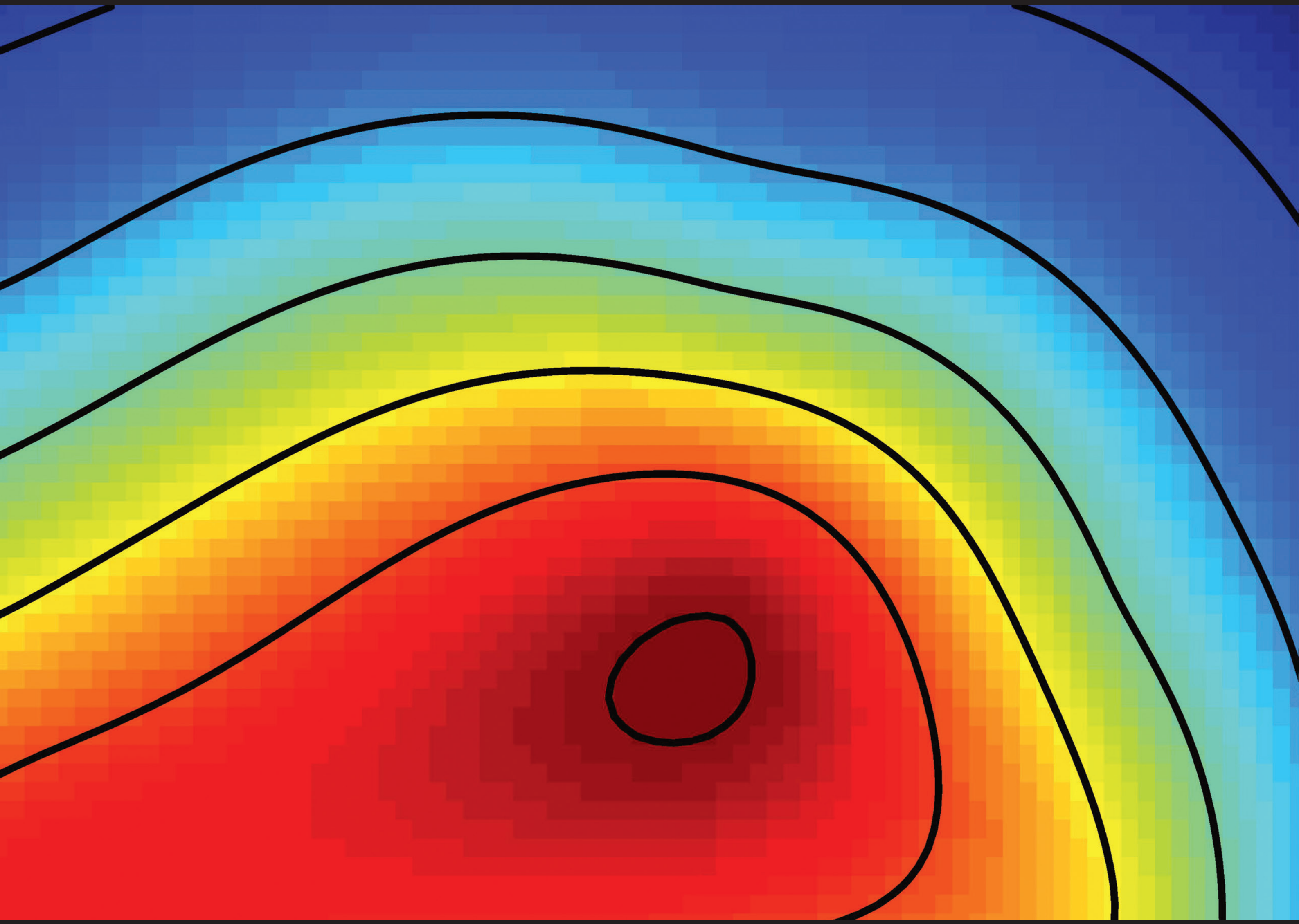

WILEY

www.aging-cell.com

Aging Cell

Open Access

### EDITOR-IN-CHIEF

**Monty Montano**
*Harvard Medical School*
*221 Longwood Ave*
*Boston, MA USA*
*Tel: 617-525-9046*
*Academic email: mmontano@bwh.harvard.edu*
*Journal email: mmontano@wiley.com*

### EDITORIAL BOARD

*Carlos Andres Aguilar*, University of Michigan, USA
*Sunil Ahuja*, UT Health San Antonio, USA
*Moran Amit*, MD Anderson Cancer Center, USA
*Roz Anderson*, University of Wisconsin, USA
*Salvador Aznar Benitah*, ICREA and Institute for Research in Biomedicine, Spain
*Shalender Bhasin*, Harvard Medical School, USA
*Morgan Carlson*, Regulus Therapeutics, USA
*Tamir Chandra*, MRC Human Genetics Unit, University of Edinburgh, UK
*Claudia Chini*, Mayo Clinic, USA
*Eduardo Chini*, Mayo Clinic, USA
*Rosaly Correa-de-Araujo*, National Institute on Aging (NIA/NIH), *USA*
*Rafael De Cabo*, National Institute on Aging, USA
*Alan Cohen*, Columbia University, USA
*Lynne S. Cox*, University of Oxford, UK
*Luigi Ferrucci*, National Institute on Aging (NIA/NIH), USA
*Judith Frydman*, Stanford University, USA
*David Furman*, Stanford University and Buck Institute, USA
*Gregory C. Gibson*, Georgia Tech, USA
*David Glass*, Regeneron Pharmaceuticals, USA
*Ann V. Griffith*, UT Health San Antonio, USA
*Yosef Gruenbaum*, The Hebrew University of Jerusalem, Israel
*Giovanni Guaraldi*, University of Modena, Italy
*Ming Guo*, University of California, USA
*Jing-Dong Jackie Han*, Chinese Academy of Sciences, China
*Curtis Henry*, Emory University, USA
*Pidder Jansen-Dürr*, Institute for Biomedical Aging Research, Austria
*Heinrich Jasper*, Buck Institute, USA
*Moustapha Kassem*, University Hospital of Odense, Denmark
*Deok-Ho Kim*, Johns Hopkins University, USA
*James Kirkland*, Mayo Clinic, USA
*Gary Krishnan*, Eli Lilly and Company, USA
*George Kuchel*, University of Connecticut, USA
*Alan Landay*, Rush University, USA
*David Le Couteur*, University of Sydney, Australia
*Seung-Jae V. Lee*, Korea Advanced Institute of Science and Technology, South Korea
*Guang-Hui Liu*, Chinese Academy of Sciences, China
*Lei Liu*, Harvard Medical School, USA
*Michael Lustgarten*, Tufts University, USA
*Vincent C. Marconi*, Emory University, USA
*Vincent Monnier*, Case Western Reserve University, USA
*Janko Nikolich-Zugich*, University of Arizona, USA
*Thomas Nystrom*, Goteborg University, Sweden
*Salvatore Oddo*, University of Messina, Italy
*Sang Chul Park*, Well Aging Research Center, Korea
*Karol Pencina*, Harvard Medical School, USA
*Domenico Praticò*, Temple University, USA
*Daniel Promislow*, University of Washington, USA
*Blake B. Rasmussen*, UT Health San Antonio, USA
*Lene Juel Rasmussen*, University of Copenhagen, Denmark
*R. Keith Reeves*, Duke University, USA
*Paul D. Robbins*, University of Minnesota, USA
*Noga Ron-Harel*, Technion – Israel Institute of Technology, Israel
*Naruhiko Sahara*, National Institute of Radiological Sciences, Japan
*Jason Sanders*, Vertex Pharmaceuticals, USA
*Stefan Schreiber*, Christian-Albrechts-Universität, Germany
*Colin Selman*, Unversity of Glasgow, UK
*Paul Shiels*, University of Glasgow, UK
*Liou Y. Sun*, University of Alabama at Birmingham
*Joseph Takahashi*, UT Southwestern and Howard Hughes Medical Institute
*LaDora Thompson*, Boston University, USA
*Xiao-Li Tian*, Institute of Molecular Medicine, Peking University,China
*Derek Alan Wainwright*, Loyola University, USA
*Jeremy D. Walston*, Johns Hopkins University, USA
*Ryan White*, Regeneron Pharmaceuticals, USA
*Matthew Yousefzadeh*, Columbia University, USA
*Chuankai (Kai) Zhou*, The Buck Institute, USA

Aging Cell

Open Access

### AIMS AND SCOPE

*Aging Cell* is an Open Access publication addressing the fundamental issues in the biology of aging. The journal covers all areas of geroscience, highlighting research that reports the mechanistic aspects of the aging process, as well as the links between aging and age-related disease.
*Aging Cell* considers for publication, articles on the following topics:

- **Basic molecular and cellular aging processes**
- **Hallmarks of aging**
- **Disease conditions informed by aging science**
- **Clinical trials based on geroscience leads**
- **Academic-Industry studies in geroscience**
- **Basic and clinical biomarkers of aging**
- **New theories of aging and comparative studies**

**OUT OF SCOPE:** Purely descriptive studies will not be considered **unless** they report major new advances within the field of study. Papers that focus on the **pathogenesis of a specific age-related disease** will **only** be considered if they offer new insights into fundamental questions in aging biology.

***Open Access and Copyright***. All articles published by *Aging Cell* are fully open access: immediately freely available to read, download and share. All *Aging Cell* articles are published under the terms of the Creative Commons Attribution License which permits use, distribution and reproduction in any medium, provided the original work is properly cited, and allows the commercial use of published articles.

Copyright on any research article in a journal published by *Aging Cell* is retained by the author(s). Authors grant Wiley a license to publish the article and identify itself as the original publisher. Authors also grant any third party the right to use the article freely as long as its integrity is maintained and its original authors, citation details and publisher are identified. Further information about open access license and copyright can be found on http://www.wileyopenaccess.com/details/content/12f25db4c87/Copyright--License.html.

***Use by commercial 'for-profit' organisations***. Use of Wiley Open Access articles for commercial, promotional, or marketing purposes requires further explicit permission from Wiley (corporatesales@wiley.com) and will be subject to a fee. Commercial purposes include:

- Copying or downloading of articles, or linking to such articles for further redistribution, sale or licensing;
- Copying, downloading or posting by a site or service that incorporates advertising with such content;
- The inclusion or incorporation of article content in other works or services (other than normal quotations with an appropriate citation) that is then available for sale or licensing, for a fee (for example, a compilation produced for marketing purposes, inclusion in a salespack);
- Use of article content (other than normal quotations with appropriate citation) by forprofit organisations for promotional purposes;
- Linking to article content in e-mails redistributed for promotional, marketing or educational purposes;
- Use for the purposes of monetary reward by means of sale, resale, licence, loan, transfer or other form of commercial exploitation such as marketing products;
- Print reprints of Wiley Open Access articles can be purchased from corporatesales@wiley.com.

*Disclaimer*. The Publisher, the Anatomical Society and Editors cannot be held responsible for any errors in or any consequences arising from the use of information contained in this journal. The views and opinions expressed do not necessarily reflect those of the Publisher, the Anatomical Society or Editors; neither does the publication of advertisements constitute any endorsement by the Publisher, the Anatomical Society, Editors, or Authors of the products advertised.

Wiley Open Access articles posted to repositories or websites are without warranty from Wiley of any kind, either express or implied, including, but not limited to, warranties of merchantability, fitness for a particular purpose, or non-infringement. To the fullest extent permitted by law Wiley disclaims all liability for any loss or damage arising out of, or in connection with, the use of or inability to use the content.

Cover legend: The image illustrates the effects of percent yeast and diet concentration on egg production rate (eggs/day), from Fanson *et al.*, 2009, pp. 514–523.
